# Magnetic Nanoparticles of Zinc/Calcium Ferrite Decorated with Silver for Photodegradation of Dyes

**DOI:** 10.3390/ma12213582

**Published:** 2019-10-31

**Authors:** Ricardo J. C. Fernandes, Carlos A. B. Magalhães, Carlos O. Amorim, Vítor S. Amaral, Bernardo G. Almeida, Elisabete M. S. Castanheira, Paulo J. G. Coutinho

**Affiliations:** 1 Centre of Physics (CFUM), University of Minho, Campus de Gualtar, 4710-057 Braga, Portugal; rica45fernandes@gmail.com (R.J.C.F.); pg30870@alunos.uminho.pt (C.A.B.M.); bernardo@fisica.uminho.pt (B.G.A.); ecoutinho@fisica.uminho.pt (E.M.S.C.); 2 Physics Department and CICECO, University of Aveiro, Campus de Santiago, 3810-193 Aveiro, Portugal; amorim5@ua.pt (C.O.A.); vamaral@ua.pt (V.S.A.)

**Keywords:** magnetic nanoparticles, zinc/calcium ferrite, silver decorated nanoparticles, photodegradation, textile dyes

## Abstract

Magnetic nanoparticles of zinc/calcium ferrite and decorated with silver were prepared by coprecipitation method. The obtained nanoparticles were characterized by UV/Visible absorption, XRD, TEM and SQUID. The mixed zinc/calcium ferrites exhibit an optical band gap of 1.78 eV. HR-TEM imaging showed rectangular nanoplate shapes with sizes of 10 ± 3 nm and aspect ratio mainly between 1 and 1.5. Magnetic measurements indicated a superparamagnetic behavior. XRD diffractograms allowed a size estimation of 4 nm, which was associated with the nanoplate thickness. The silver-decorated zinc/calcium ferrite nanoparticles were successfully employed in the photodegradation of a model dye (Rhodamine B) and industrial textile dyes (CI Reactive Red 195, CI Reactive Blue 250 and CI Reactive Yellow 145). The nanosystems developed exhibited promising results for industrial application in effluent photoremediation using visible light, with the possibility of magnetic recovery.

## 1. Introduction

Nowadays, one of the major problems worldwide is water pollution. Considering population growth and the consequent increase in industrialization, pollution levels in water resources have grown dramatically. The textile industry appears as one of the most polluting sectors worldwide. It deals daily with millions of liters of water, leaving an associated trace of color in its effluents, which represents one of the main problems of this industrial sector [1,2].

Recently, several works have drawn attention to nanotechnology for environmental applications and specifically to magnetic nanoparticles, some of them being capable of degrade textile dyes by photodegradation [3].

For many years, titanium dioxide has been used as the photocatalyst of excellence. However, its large band gap of 3.2 eV decreases its applicability, by only degrading in the presence of UV light [4]. Lower energy radiation (e.g. in the visible spectrum) can be used if the bandgap of the semiconductor is reduced. In this context, zinc ferrites appear as a promising semiconductor (band gap of 1.9 eV), promoting photodegradation of dyes under visible light, as demonstrated in recent studies [4]. However, focusing on photoremediation of industrial effluents, the magnetic properties of the nanoparticles must be improved, to allow magnetic recovery and reuse of the photocatalysts. Biocompatibility is also a feature to pursue, considering applications in the photoremediation of water natural resources. The incorporation of calcium in the nanoparticles composition, giving mixed zinc/calcium ferrite nanoparticles, allows obtaining stable ferrites with enhanced biocompatibility and magnetic properties [5,6].

One of the main limitations for the application of ferrite nanoparticles is their low separation efficiency of electrons and holes, which leads to a much lower photocatalytic activity comparing to titanium dioxide [4]. It has been shown that the deposition of a noble metal at nanoparticle surface increases the separation rate of electrons and holes, promoting the transfer to the interfacial load [7,8]. In the present work, silver was used to cover the nanoparticles surface [4,7,8]. Since the rate of recombination of ferrites is high, reducing their photocatalytic activity, the incorporation of silver reduces rapid recombination of the generated electron/hole pairs, increasing the formation of reactive species and allowing an enhanced photocatalytic activity.

In this work, mixed zinc/calcium ferrite nanoparticles decorated with silver clusters were tested as photodegradation agents for textile reactive azo dyes, namely Reactive Red 195 (“Red”), Reactive Blue 250 (“Blue”) and Reactive Yellow 145 (“Yellow”). These dyes have a general structure R − N = N − R’ (Table 1) and are the most used class in industrial dyeing processes, being generally persistent in final industrial effluents [9,10]. Rhodamine B (structure in Table 1) was also used as model dye for comparison, due to its well-known photophysical properties [11,12] and wide use in photodegradation assays [13,14,15]. 

The proposed nanoparticles are advantageous for waste water treatment, as the incorporation of Zn cations may promote antimicrobial activity and the presence of calcium in the ferrite structure favors biocompatibility of the nanoparticles. Studies in cell lines have shown that, for a 100 μg/mL concentration of nanoparticles, zinc ferrite allows 70.5% of cell viability at 24 h, while calcium ferrite allows 90.6% of cell viability for the same concentration and time of exposure. For comparison, using cobalt ferrite nanoparticles, 80% of cell viability was observed but with only 20 μg/mL of nanoparticles [16]. Therefore, enhanced biocompatibility is expected by inclusion of calcium in zinc ferrites. The nanosystems here developed show promising results for industrial application in effluent photoremediation.

## 2. Materials and Methods

### 2.1. Nanoparticles Preparation

#### 2.1.1. Zinc/Calcium Ferrite Nanoparticles

Zinc/calcium ferrite nanoparticles were prepared through a coprecipitation method in reflux conditions, adapting a previously described procedure by Cao et al. [4]. First, 1.082 g of iron (III) chloride hexahydrate, 0.219 g of zinc acetate and 0.158 g of calcium acetate were dissolved in 200 mL of ultrapure water Milli-Q grade (MilliporeSigma, St. Louis, MO, USA). After dissolution, 1 mL of oleic acid and 1.198 g of urea were added to the solution. After complete dispersion, the solution was refluxed vigorously for at least 3 h. 

For purification, the obtained sample was washed several times with absolute ethanol and ultrapure water, by magnetic decantation and centrifugation (14,000 g). The mixed ferrite nanoparticles were dried for 12 h at 90 °C. To improve crystallinity, the zinc/calcium ferrite nanoparticles were calcined at 400 °C for 30 min. 

#### 2.1.2. Zinc/Calcium Ferrites Decorated with Silver Clusters 

The as-prepared mixed ferrite nanoparticles (either calcined or non-calcined) were dispersed in 100 mL of ethylene glycol. Next, 0.160 g of silver nitrate were dissolved in 20 mL of ultrapure water and added to the previous dispersion. This solution was refluxed for 30 min. The products were separated by magnetic decantation and centrifugation (14,000 g) and washed repeatedly with absolute ethanol. The nanoparticles were dried for 12 h at 90 °C.

### 2.2. Structural Characterization

#### 2.2.1. Transmission Electron Microscopy (TEM)

TEM images of nanoparticles were acquired using a Transmission Electron Microscope JEOL 2100 (JEOL USA Inc., Peabody, MA, USA) operating at 200 kV coupled to an Electron Dispersive X-Ray Spectroscopic analyzer (EDS). The solutions were sonicated in ethanol and dropped onto a TEM grid (copper 400 mesh with a carbon film). TEM images were processed using ImageJ 1.52p software (National Institutes of Health (NIH), Bethesda, MD, USA). The size of each particle was determined by equalizing its area with the area of a circle. However, this is a crude approximation for the type of particles observed in TEM images. Thus, an additional estimation was made by inscribing rectangular shapes on each particle. The ratio of resulting side lengths of the obtained rectangles was used as an estimation of the aspect ratio.

#### 2.2.2. X-Ray Diffraction (XRD) 

X-Ray Diffraction (XRD) analyses were performed using a conventional Philips PW 1710 (Royal Philips, Amsterdam, The Netherlands) diffractometer, operating with CuK_α_ radiation, in a Bragg-Brentano configuration. 

#### 2.2.3. Magnetic Measurements

Magnetization measurements were done in a MPMS3 SQUID magnetometer (Quantum Design Inc., San Diego, CA, USA). The hysteresis cycles (magnetization versus magnetic field) of the samples were measured in the convenient field range for each sample, with a possible maximum +/−70 kOe (+/−7 Tesla). The measurement method was by DC extraction or VSM oscillation at a frequency of 14 Hz. A specific magnetic field correction for the trapped flux in the superconducting coil was made achieving an accuracy of residual less than 2 Oe.

### 2.3. Photodegradation Assays

To evaluate the photocatalytic activity of the as-prepared nanoparticles, a home-built irradiation apparatus was used. The setup incorporates a 200 W Xenon Arc Lamp (L.O.T.-Oriel GmbH & Co. KG, Darmstadt, Germany), a 400 nm long pass filter (Thorlabs Inc., Newton, NJ, USA) to isolate the visible spectrum radiation, and a sample cuvette holder. Aqueous solutions of Rhodamine B (40 mg/L) and of textile dyes, C. I. Reactive Red 195 (“Red”), C. I. Reactive Blue 250 (“Blue”) and C. I. Reactive Yellow 145 (“Yellow”) (80 mg/L) were assayed for 2.5 h. In the first 30 min, the nanoparticles were added to the solution, in constant stirring, under dark. After this initial time, the sample solution was exposed to light under magnetic stirring, and aliquots were taken at 0, 5, 10, 15, 30, 60, 90 and 120 min. The photocatalyst content of each aliquot was removed by centrifugation and the absorption spectra were recorded in a Shimadzu UV-3600 Plus UV-Vis-NIR (Shimadzu Corporation, Kyoto, Japan) spectrophotometer. 

## 3. Results and Discussion

### 3.1. Nanoparticles Characterization

#### 3.1.1. Absorption Spectra

**Figure 1:**
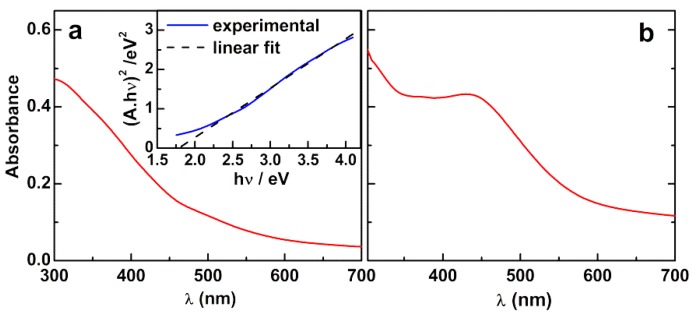
UV-Visible absorption spectra of aqueous dispersions of (**a**) zinc/calcium ferrite nanoparticles and (**b**) silver-decorated nanoparticles.

Figure 1 displays the UV-Visible absorption spectra of aqueous dispersions of mixed zinc/calcium ferrite nanoparticles and Ag-decorated zinc/calcium ferrite nanoparticles. 

The spectrum of the mixed zinc/calcium ferrite nanoparticles in Figure 1a allows the determination of the optical band gap, using a Tauc plot (Equation (1)),

(1) $${\left(\alpha h\nu \right)}^{n}\propto \;\left(h\nu -E_{g}\right)$$

where α is the absorption coefficient (proportional to the absorbance), n is an exponent that depends on the nature of the transition (being n = 2 for a direct semiconductor and n = 1/2 for an indirect one) and E_g_ is the optical band gap [17]. A band gap of 1.78 eV (n = 2) was estimated from the intercept of inset of Figure 1a, in agreement with the value of 1.90 eV reported by Kim et al. for calcium ferrite nanoparticles [18], as well as for zinc ferrite [4].

Comparing the nanoparticles without and with silver (Figure 1), it can be observed the characteristic local surface plasmon resonance (LSPR) band of silver nanoparticles around 435 nm, within the range of values previously reported [19]. 

#### 3.1.2. X-Ray Diffraction (XRD) Measurements

**Figure 2:**
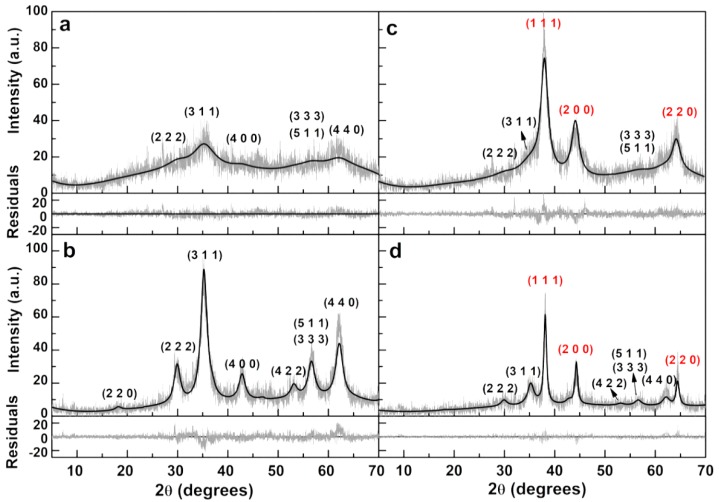
XRD diffractograms of zinc/calcium ferrite nanoparticles: (**a**) Non-calcined zinc/calcium ferrite; (**b**) calcined zinc/calcium ferrite; (**c**) non-calcined zinc/calcium ferrite decorated with silver; (**d**) calcined zinc/calcium ferrite decorated with silver. Gray lines: Experimental patterns; black lines: fitted patterns. Miller indices: Black: zinc/calcium ferrite; Red: Silver.

The calcination process allows an improvement in crystallinity and magnetic properties of the nanoparticles, which is essential for their recovery at the end of the irradiation procedure, enabling the possibility to recycle and reuse the nanoparticles [20,21]. XRD analysis revealed a strongly amorphous background for the non-calcined nanoparticles (Figure 2a). Upon calcination, several well defined diffraction peaks are observed (Figure 2b). Using FullProf software (version 5.8, J. Rodríguez-Carvajal, Lab. Léon Brillouin, Gif sur Yvette, France) [22], Rietveld analysis of calcined zinc/calcium ferrite diffractogram was performed, by adapting CIF file number 2300615 (partially inverted cubic spinel phase, space group Fd$\bar{3}$m), corresponding to zinc ferrite, to have 50% occupation with Zn and 50% with Ca at the zinc lattice sites. Bulk zinc ferrite has a direct spinel structure. However, it was found that in nanoparticles the degree of inversion, i, increases with the decrease of nanoparticle size [23], with a corresponding enhancement of magnetic properties. Recently, it was reported that mixed zinc/calcium ferrites adopt an inverted spinel structure [24]. Thus, an inverted spinel structure is considered, in which the A^2+^ ions in octahedral sites are 50% distributed between zinc and calcium: (Fe)^Td^(FeZn_0.5_Ca_0.5_)^Oh^O_4_. A reasonable value of R_F_ = 4.35 (Table 2) was obtained, indicating that the assumed crystal structure is compatible with the XRD results, since all the corresponding diffraction peaks are observed and have nearly the calculated intensities (Figure 2b). 

The average size that results from Debye-Scherrer equation, as implemented by FullProf suite [22], is 3.97 nm and the lattice constant is 8.425 Å. The lattice constant for bulk ZnFe_2_O_4_ is 8.443 Å [25], but values down to 8.411 Å using thermal decomposition method [25], and 8.391 Å using microwave synthesis [26], and up to 8.47 Å when using coprecipitation method [23], were reported. Calcium ferrite nanoparticles in the spinel crystallographic form and using co-precipitation methods have lattice constants between 8.34 Å [27] and 8.37 Å [28]. Thus, the lattice constant of the here obtained zinc/calcium mixed ferrite lies between the corresponding single ferrite phases. Rietveld analysis on the non-calcined sample (Figure 2a) is compatible with 1.1 nm size. Both calcined and non-calcined samples were coupled with metallic silver. Its presence is confirmed in the corresponding XRD diffractograms presented in Figure 2c,d, respectively. The Rietveld analysis using an additional phase corresponding to silver (CIF 9008459) allows an estimation of size of the coupled silver nanoparticles, as well as of their amount in each sample. A summary of the Rietveld analysis of all samples is shown in Table 2 and the resulting weight percentages of ferrite and silver are indicated in Table 3.

It can be observed (Table 3) that the Ag coupled calcined nanoparticles exhibit a lower silver content, indicating that silver exhibits more affinity for the non-calcined amorphous nanoparticles. However, the significant enhancement of the crystalline structure of the nanoparticles with calcination is determinant in obtaining suitable magnetic properties for environmental applications.

#### 3.1.3. Transmission Electron Microscopy (TEM)

**Figure 3:**
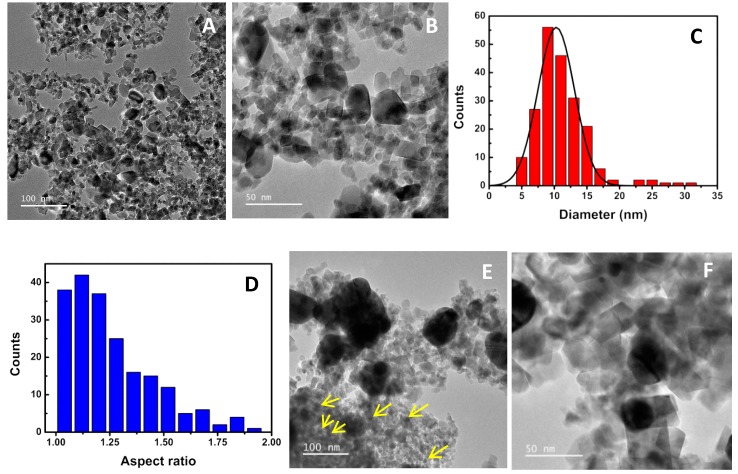
TEM images of the synthesized nanoparticles. (**A**,**B**): Zinc/calcium ferrite nanoparticles; (**C**): Particle size histogram of image (**B**) and fitting to a Gaussian distribution; (**D**): Aspect ratio histogram of particles in image (**B**); (**E**,**F**): Zinc/calcium ferrite nanoparticles containing silver.

TEM images of the calcined zinc/calcium ferrite nanoparticles (Figure 3A,B) revealed generally rod-like or prismatic shapes, with a size distribution of 10 ± 3 nm (Figure 3C), obtained considering circles with the same area of each of the 206 particles that were manually delimited. Assuming instead a rectangular shape (ImageJ bonding rectangle), sizes of longer and shorter sides are, respectively, 12 ± 3 nm and 9.8 ± 3 nm, with a broad aspect ratio distribution between 1.04 and 2 (Figure 3D).

The difference in size from XRD estimation might be related to a nanoplate-like structure, already reported for zinc ferrite [4], where its thickness corresponds to the size determined by XRD (the nanoplates are lying down, so that only their thickness contributes to the amount of lattice planes that define the X-ray diffraction signal). EDX analysis (average of 5 measurements) allowed obtaining a ratio of Zn/Fe atomic percentages of 26.7%, in accordance to what was expected for Zn_0.5_Ca_0.5_Fe_2_O_4_ (Zn/Fe ratio of 25%). 

TEM images of zinc/calcium ferrite nanoparticles decorated with silver clusters (Figure 3E,F) show the additional appearance of more spherical shapes (marked on Figure 3E,F) and also of agglomerates of these spherical particles. These have sizes of 9.4 ± 1 nm and correspond to the silver content of the prepared sample, being compatible with the size estimation obtained from XRD. EDX analysis estimated an atomic silver percentage of 21%, slightly smaller than the determined by XRD.

The position of the LSPR band (Figure 1B) depends on the size, shape and refractive index of the medium surrounding the silver nanoparticle. Considering silver nanospheres in water, for which citrate was used as stabilizing agent, the plasmon band for 10 nm size should appear at 398 nm [29]. However, in this case, the deposited silver particles are expected to be either nanodisks or half-spheres, with one side surrounded by the Zn/Ca mixed ferrite and the other side facing an aqueous environment. An increase in refractive index is expected to induce a red shift in the plasmon band. The refractive index of ZnFe_2_O_4_ in 400–500 nm region is above 2 [30], so that a significant red shift from 398 nm is expected. Also, the shape and its aspect ratio have a pronounced effect on the LSPR band position, with nanodisks of 10 nm diameter and 2 nm height showing two plasmon bands, one at ~420 nm and the other, more intense, at ~560 nm [19]. Thus, the observed plasmon band at 435 nm is not incompatible with the ~10 nm size determined by TEM and XRD.

**Figure 4:**
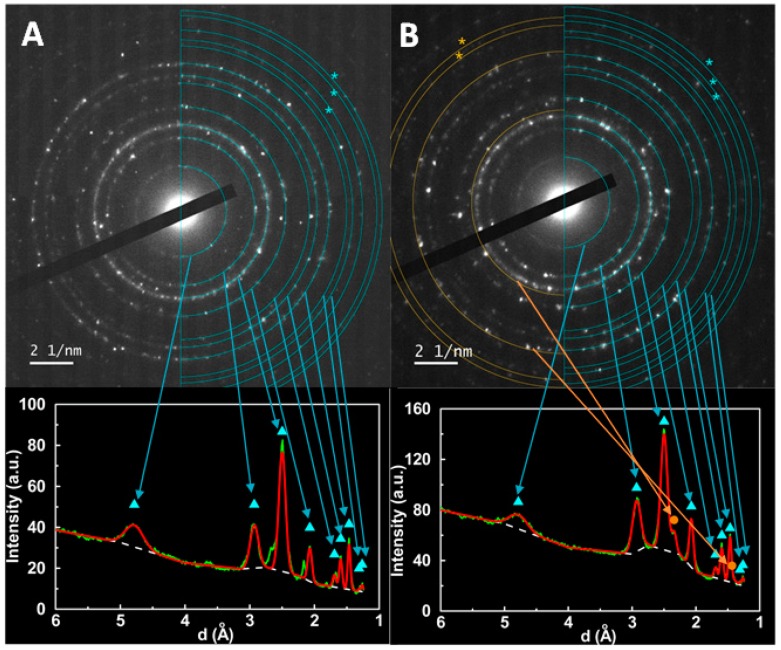
TEM SAED images of the synthesized zinc/calcium ferrite nanoparticles, without (**A**) and with silver (**B**). Below each image, a radial profile together with a fit is represented, considering the diffraction lines of zinc/calcium ferrite (marked by cyan triangles) and silver (marked by orange circles). Rings marked by asterisks pass by additional diffraction spots, but did not define a complete circle, not being possible to use in the radial profile.

Small area electron diffraction (SAED) images of zinc/calcium ferrite samples without (Figure 4A) and with silver (Figure 4B) show diffraction spots that can be associated with ferrite phase (cyan rings) and silver (orange rings), as follows. The circular profile of the images was obtained using the radial profile ImageJ plugin and fitted to a sum of Gaussian functions, with variable intensities and halfwidths, but with central positions defined by d-spacing values calculated from the diffraction crystal planes, corresponding to either spinel or fcc crystal structures by optimizing only the lattice constants of each phase. This procedure allowed localization of the rings indicated in Figure 4, with lattice constants of 8.286 Å for zinc/calcium ferrite and 4.055 Å for silver. The diffraction planes corresponding to the peaks are (1 1 1); (2 2 0); (3 1 1); (4 0 0); (4 2 2); (3 3 3) + (5 1 1); (4 4 0); (6 2 0) and (5 3 3) for zinc/calcium ferrite, and (1 1 1) and (2 2 0) for silver. Additional diffraction spots, not used in the circular profile, can be identified to the (4 4 4) + (7 1 1); (5 5 1); (6 4 2) and (7 3 1) + (5 5 3) diffraction planes of zinc/calcium ferrite and (3 1 1) and (2 2 2) of silver, being marked with * in Figure 4.

### 3.2. Magnetic Properties

**Figure 5:**
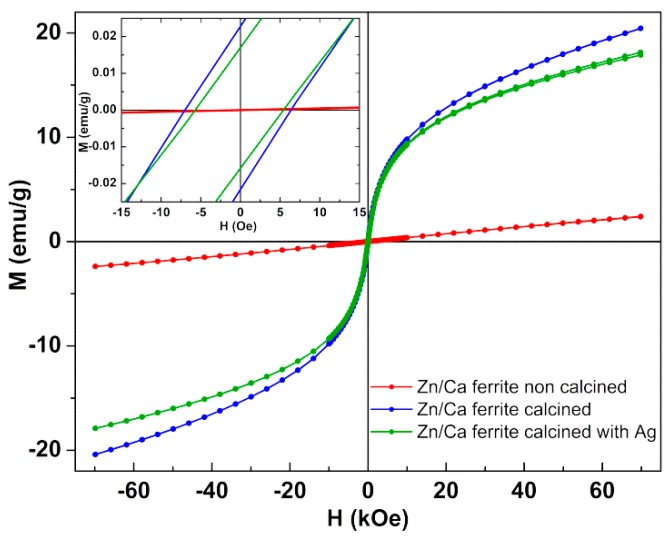
Magnetization hysteresis loop of zinc/calcium ferrite nanoparticles (non-calcined and calcined) and zinc/calcium ferrite nanoparticles coated with silver at room temperature. Inset: Enlargement of the loop in the low field region.

The magnetic properties of the prepared zinc/calcium ferrite nanoparticles, with and without silver coating (Figure 5) were characterized by measuring their magnetic hysteresis loop, which shows the relationship between the induced magnetic moment and the applied magnetic field (H). The calcined nanoparticles present a superparamagnetic behavior, as the ratio between remnant magnetization (M_r_) and maximum magnetization (M_s_) is below 0.1 (Table 4). If below 0.1, this ratio indicates that more than 90% of the magnetization is lost upon the removal of the applied magnetic field [31,32]. The very low maximum magnetization of the non-calcined nanoparticles is justified by their highly amorphous nature, as verified by XRD. With calcination, the maximum magnetization increases ten times (Figure 5), maintaining a low coercivity.

The saturation magnetization of zinc ferrite nanoparticles depends strongly on the preparation method, with a reported value of M_s_ = 1.638 emu/g for ZnFe_2_O_4_ prepared by microwave combustion method [33] and of M_s_ = 13.11 emu/g for zinc ferrite obtained by sol-gel [24]. This is because zinc is a non-magnetic cation, its position in tetrahedral and octahedral lattice sites strongly influencing the maximum magnetization [33]. Here, the calcined Zn/Ca mixed ferrite nanoparticles present a magnetization slightly higher than the one reported for Zn_0.75_Ca_0.25_Fe_2_O_4_ and lower than that of Zn_0.5_Ca_0.5_Fe_2_O_4_ produced by sol-gel method (M_s_ = 31.31 emu/g) [24]. The mass content of silver estimated from the decrease in maximum magnetization (in emu/g) of the zinc/calcium ferrite is 27%, roughly in accordance with the value estimated from XRD. 

The reasonable magnetization of these nanoparticles and the superparamagnetic behaviour point to their promising use in water remediation, despite superparamagnetism is not mandatory for this application. For recycling the particles, these must have a reasonable maximum magnetization, which is here the case of calcined nanoparticles. By application of an external magnetic field (e.g., a magnet), the nanoparticles are readily attracted. Therefore, by using a magnetic filter, for example, the nanoparticles can be removed from the effluent and recovered for subsequent reuse. Moreover, Zn_0.5_Ca_0.5_Fe_2_O_4_ nanoparticles were reported to be biocompatible and not hemolytic, in concentrations up to 10 mg/mL, whereas CaFe_2_O_4_ nanoparticles were shown to be hemolytic in the same concentration range [24].

### 3.3. Photodegradation Assays

#### 3.3.1. Assays in Neat Dye Solutions

**Figure 6:**
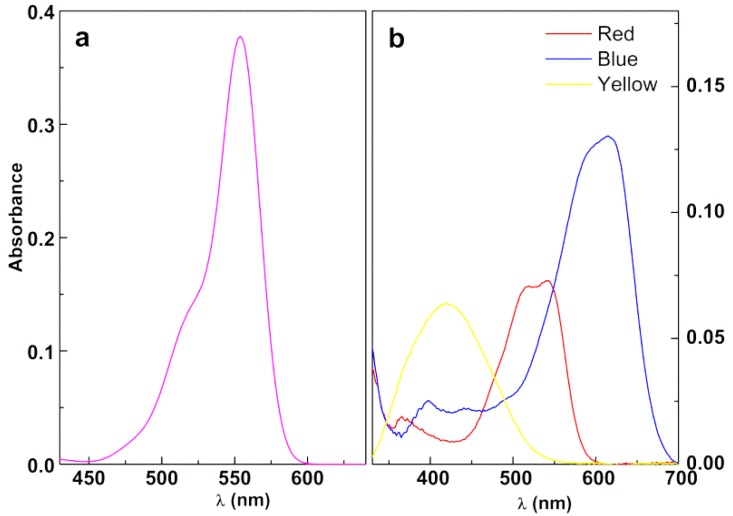
UV-Visible absorption spectra of aqueous solutions of (**a**) Rhodamine B (40 mg/L) and (**b**) industrial reactive dyes (80 mg/L). Red: C.I. Reactive Red 195; Blue: C.I. Reactive Blue 250; Yellow: C.I. Reactive Yellow 145.

Figure 6 exhibits the absorption spectra of Rhodamine B and industrial textile dyes solutions. As expected, the absorption spectrum of each dye exhibits a main band characteristic of the complementary of each color.

**Figure 7:**
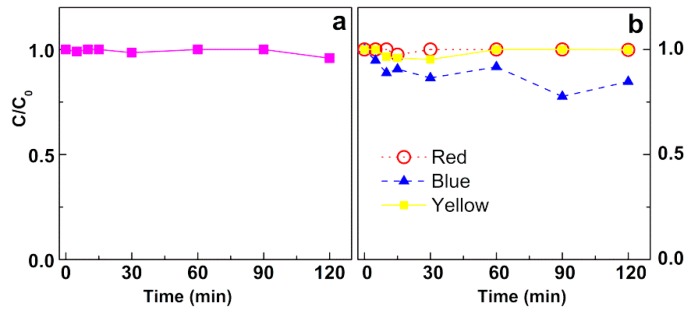
Photodegradation assays of solutions of (**a**) Rhodamine B (40 mg/L) and (**b**) industrial reactive dyes (80 mg/L) in the absence of magnetic nanoparticles. Red: C.I. Reactive Red 195; Blue: C.I. Reactive Blue 250; Yellow: C.I. Reactive Yellow 145.

A blank assay was performed for each dye solution, where it was irradiated in the absence of magnetic nanoparticles. The results of these assays are displayed in Figure 7. It can be observed a negligible degradation of the dyes (<4%) in the period of two hours, except for the reactive textile Blue dye, where a 16% degradation was observed after 120 min.

#### 3.3.2. Photodegradation of the Model Dye Using Nanoparticles

For assessment of the photocatalytic effect of the prepared nanoparticles, a model dye, Rhodamine B, was employed. No photodegradation was observed after 120 min irradiation (λ > 400 nm) using the silver-free zinc/calcium ferrite nanoparticles. However, the silver-decorated nanoparticles show efficient photodegradation capability using visible light photons (SPR-assisted photocatalytic degradation), depending on the nanoparticles concentration in solution. This enhanced photodegradation capability after Ag incorporation in Zn_0.5_Ca_0.5_Fe_2_O_4_ nanoparticles is due to enhanced charge separation owing to electron transport to Ag clusters anchored in the magnetic nanoparticles surface [34], consequently reducing the recombination probability of the photogenerated electron-hole pairs [35].

**Figure 8:**
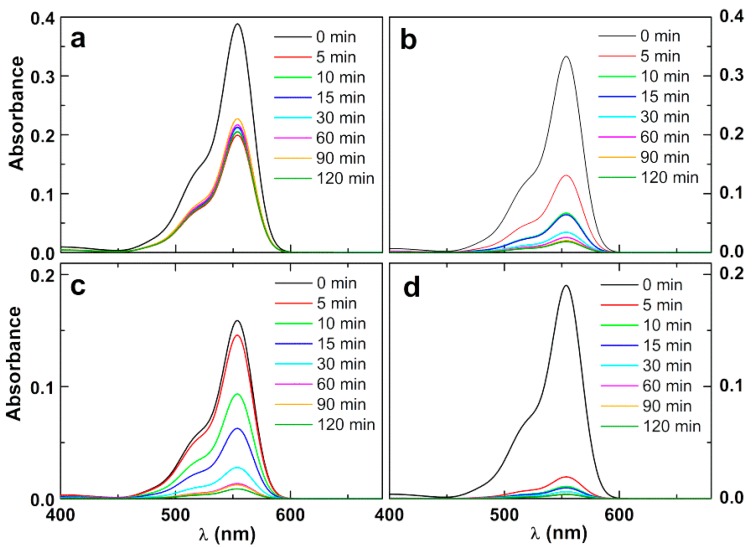
Photodegradation assays of solutions of the dye Rhodamine B (40 mg/L) by silver-decorated zinc/calcium ferrite nanoparticles (NPs), at various concentrations: absorption spectra of Rhodamine B solution at several irradiation times. (**a**) 2 mg/mL of Ag-coated calcined NPs; (**b**) 2 mg/mL of Ag-coated non-calcined NPs; (**c**) 3 mg/mL of Ag-coated calcined NPs; (**d**) 4 mg/mL of Ag-coated calcined NPs.

**Figure 9:**
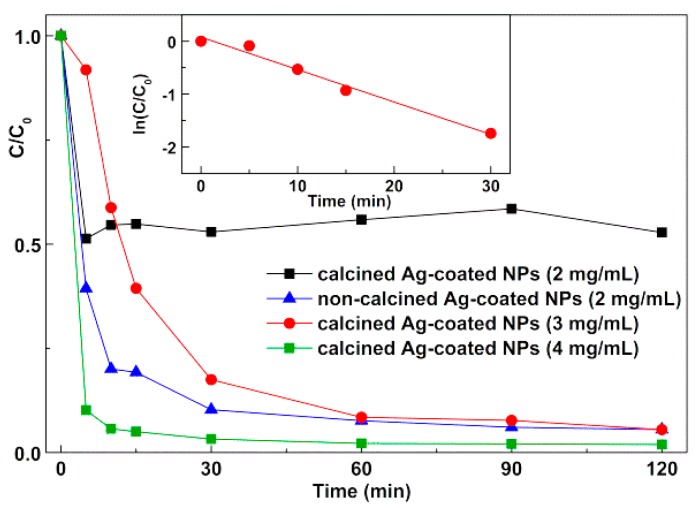
Photodegradation curves for Rhodamine B solution (40 mg/L) by silver-decorated zinc/calcium ferrite nanoparticles (NPs), at various concentrations. Inset: Plot of the pseudo-first-order kinetics for calcined Ag-coated nanoparticles 3 mg/mL (for the first 30 min).

Figure 8 shows the photodegradation of Rhodamine B using the silver-decorated zinc/calcium ferrite nanoparticles. The different absorbance of Rhodamine B solution at time zero is due to variations in dye adsorption to nanoparticles surface in the dark. It can be observed (Figure 9) that the majority of the dye is degraded in the first 30 min. For Ag-decorated calcined nanoparticles at 2 mg/mL, a very fast photodegradation occurs during the first 5 min of irradiation, followed by a stabilization. This seems to indicate that, for this sample, a colorless Rhodamine B photodegradation product gets strongly adsorbed on the particles surface, preventing further photocatalytic events. A plateau is also observed for the other samples but, as expected, it decreases as the particle concentration increases. Comparing the resulting plateau for experiments with the same particle load (2 mg/mL) of calcined or non-calcined samples, and considering that there is a significantly lower amount of silver in the calcined nanoparticles (as estimated from XRD data), it is apparent that the product adsorption occurs mainly in silver sites. 

The kinetic constants of dye photodegradation are usually estimated by applying a pseudo-first-order kinetic model (Equation (2)) [36],

(2) $$\ln\left(C/C_{0}\right)=-\mathrm{kt}$$

where k is the photodegradation rate constant (min^−1^), C_0_ is the initial concentration of the dye and C is the concentration of the dye at different irradiation times, t.

A pseudo-first-order kinetics is only verified for the first 30 min in the case of Ag-decorated nanoparticles 3 mg/mL (inset of Figure 9), corresponding to a rate constant of 0.0614 min^−1^ (R^2^ = 0.983). In the other cases, the degradation rate is very low (calcined NPs 2 mg/mL) or too fast (non-calcined NPs and calcined NPs 4 mg/mL). This rate compares well with the one obtained for Rhodamine B photodegradation with visible light using g-C_3_N_4_ (k = 0.065 min^−1^) [14] and much higher than the one recently reported in assays with an Ag_2_S-ZnS nanozised catalyst loaded on cellulose (k = 6.4 × 10^−3^ min^−1^) [37].

**Figure 10:**
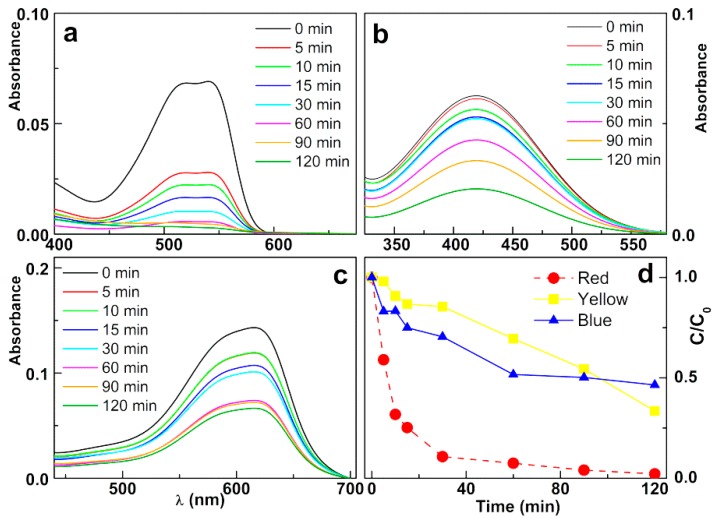
Photodegradation assays of solutions of the textile reactive dyes (80 mg/L) by silver-decorated zinc/calcium ferrite nanoparticles (2 mg/mL): absorption spectra of textile dyes solutions at several irradiation times. (**a**) Red; (**b**) Yellow (**c**) Blue; (**d**) Degradation curves. Red: C.I. Reactive Red 195; Blue: C.I. Reactive Blue 250; Yellow: C.I. Reactive Yellow 145.

In the photodegradation assays performed with the textile reactive dyes (Figure 10), both the concentrations of nanoparticles and of the dyes were the same in all assays. A very high degradation rate was achieved for the “Red” dye, reaching a percentage of degradation higher than 90%. In the case of “Yellow” and “Blue” dyes, the degradation rate is lower, reaching 65% and 40%, respectively. It should be noted that the curve of “Yellow” dye has not stabilized until 2 h of irradiation, suggesting that with a longer irradiation time, a higher degradation rate could be achieved.

**Figure 11:**
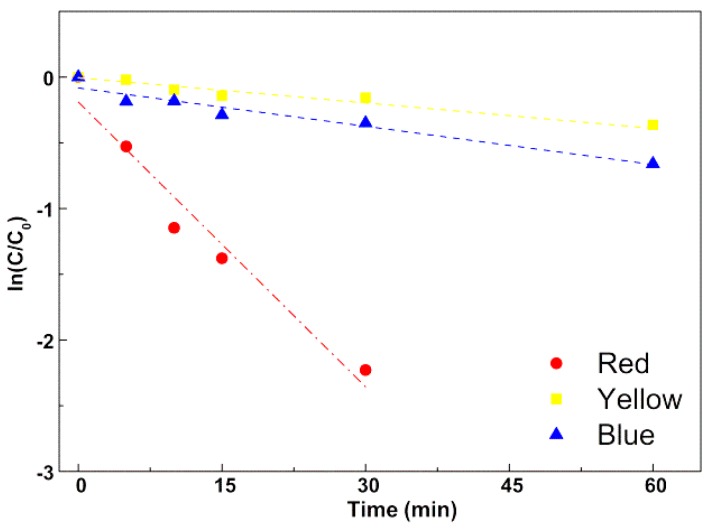
Plot of the pseudo-first-order kinetics for degradation of textile dyes. Red: C.I. Reactive Red 195; Blue: C.I. Reactive Blue 250; Yellow: C.I. Reactive Yellow 145.

The fit to a pseudo-first-order kinetic model (Figure 11) is clearly linear for the first 60 min of irradiation in the case of the dyes “Yellow” and “Blue”, and for the first 30 min in the case of “Red” dye. The rate constants for photodegradation of “Yellow”, “Blue” and “Red” are 0.0058 min^−1^ (R^2^ = 0.953), 0.0104 min^−1^ (R^2^ = 0.954), and 0.0722 min^−1^ (R^2^ = 0.959), respectively, the rate constant for “Red” dye being similar to the one determined for Rhodamine B.

It should be noted that the degradation rate strongly depends on the structure of the azo dye [38]. For ZnO photocatalysts [39], the reported value for Congo Red (CR) is 0.112 min^−1^, using UV light from a 1000 W lamp at 365 nm [39]. Here, for a comparable azo dye (CI Reactive Red 195) with two naphthalene-sulfonated groups linked to the azo moiety (as in CR dye), a comparable photodegradation rate was obtained using visible light (λ > 400 nm) and a lower lamp power (200 W). For Hispamin Black CA, another textile dye with similar structural features (two naphthalene-sulfonated moieties linked to an azo group), a lower degradation rate of 0.050 min^−1^ was achieved using oxygen peroxide oxidation and UV radiation [40]. Using a higher concentration of TiO_2_ photocatalyst (3 g/L) than the nanoparticles concentration used here (2 g/L), the dye Reactive Red 198 (Remazol Red 133, containing a naphthalene-bisulfonated moiety directly bound to the azo group) was 98% decolorized at 120 min of irradiation with UV light [41], as observed here for Reactive Red 195 and using visible radiation.

Considering our system, the inclusion of calcium ions in zinc ferrite enhances the photodegradation efficiency, as for ZnFe_2_O_4_ nanoplates with 22.7% silver load, a decrease of only 40% was observed upon 25 min of irradiation, with visible light from a 500 W Xenon lamp [4]. Therefore, the developed silver-decorated zinc/calcium ferrite nanoparticles stand out as promising nanosystems for treatment of coloured effluents from the textile industry with visible light. This is especially relevant as it has been reported that some of these dyes are toxic, mutagenic and carcinogenic compounds, being hazardous for the environment even at low concentrations [42].

## 4. Conclusions

In this work, zinc/calcium mixed ferrite nanoparticles were synthesized and decorated with silver. This innovative nanosystem was successfully employed in the photodegradation of dyes, both a model dye (Rhodamine B) and industrial textile azo dyes. The degradation efficiency using visible light was similar to the one of reported nanosystems that need UV radiation.

The developed composite nanoparticles showed to be promising for applications in photoremediation of textile effluents using visible light and subsequent magnetic recovery and reutilization, taking advantage of their magnetic properties. 

## Figures and Tables

**Table 1:** Structure of the textile dyes and model dye used for photodegradation assays.

Commercial Name	Molecular Formula	Molecular Weight (g/moL)	Molecular Structure
C.I. Reactive Blue 250 (Reactive Blue RGB)	C_27_H_23_N_5_Na_4_O_20_S_6_	1021.84	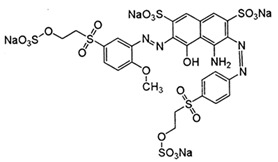
C.I. Reactive Yellow 145 (Reactive Yellow 3RS)	C_28_H_20_ClN_9_Na_4_O_16_S_5_	1026.25	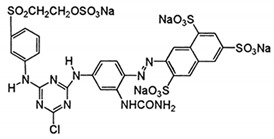
C.I. Reactive Red 195 (Reactive Red 3BS)	C_31_H_19_ClN_7_Na_5_O_19_S_6_	1136.32	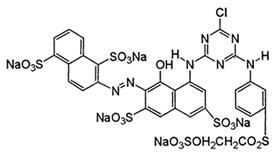
Rhodamine B	C_28_H_31_ClN_2_O_3_	479.02	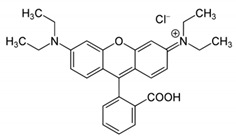

**Table 2:** Selected Rietveld analysis parameters.

Sample	O_x,y,z_ (*)	i (*)	Phase Size (nm) Lattice Constant (nm) Zn/Ca Ferrite|Ag	R_f_ Zn/Ca Ferrite|Ag	X^2^
Zn/Ca ferrite non-calcined	0.2405	1 (^+^)	1.12 | ---- 0.8425 (^+^) | ----	3.18 | ----	1.11
Zn/Ca ferrite calcined	0.2464	1 (^+^)	3.97 | ---- 0.8425 | ----	4.35 | ----	1.26
Zn/Ca ferrite non-calcined with silver	0.2405 (^+^)	1 (^+^)	1.12 (^+^) | 3.41 0.8425 (^+^) | 0.4069	3.42 | 0.95	1.32
Zn/Ca ferrite calcined with silver	0.2464 (^+^)	1 (^+^)	3.97 (^+^) | 9.90 0.8425 (^+^) | 0.4078	9.81 | 2.91	1.13

(*) Values in CIF file 2300615 are O_x,y,z_ = 0.2535 and i = 0.62; (^+^) fixed value.

**Table 3:** Estimated percentage of silver in the nanoparticles obtained by XRD.

Nanoparticles	Zn_0.5_Ca_0.5_Fe_2_O_4_ (%)	Ag (%)
Zn_0.5_Ca_0.5_Fe_2_O_4_ non-calcined	100	-
Zn_0.5_Ca_0.5_Fe_2_O_4_ calcined	100	-
Ag@Zn_0.5_Ca_0.5_Fe_2_O_4_ non-calcined	57.8	42.2
Ag@Zn_0.5_Ca_0.5_Fe_2_O_4_ calcined	66.4	33.6

**Table 4:** Coercive field (H_c_), saturation magnetization (M_s_), remnant magnetization (M_r_) and ratio M_r_/M_s_ for zinc/calcium ferrites at room temperature.

-	H_c_ (Oe)	M_s_ (emu/g)	M_r_ (emu/g)	M_r_/M_s_
Zn_0.5_Ca_0.5_Fe_2_O_4_ non-calcined	1.8	2.41	8 × 10^−5^	3 × 10^−5^
Zn_0.5_Ca_0.5_Fe_2_O_4_ calcined	7.5	20.45	0.022	1 × 10^−3^
Silver coated Zn_0.5_Ca_0.5_Fe_2_O_4_ calcined	5.3	18.14	0.016	9 × 10^−4^
